# Bidirectional mitochondrial transfer in the osteoimmune microenvironment: mechanisms and therapeutic perspectives

**DOI:** 10.3389/fendo.2026.1978936

**Published:** 2026-10-19

**Authors:** Lei Huang, Xin Chen, Yuqin Guo, Fang Guo, Yanfan Zhang, Xuanyi Li, Zhili Dong, Xiaoxing Kou

**Affiliations:** 1 Hospital of Stomatology, Guanghua School of Stomatology, Sun Yat-sen University, Guangzhou, China; 2 Guangdong Provincial Key Laboratory of Stomatology, Sun Yat-sen University, Guangzhou, China; 3 South China Center of Craniofacial Stem Cell Research, Guanghua School of Stomatology, Sun Yat-sen University, Guangzhou, China; 4 Department of Temporomandibular Joint, School and Hospital of Stomatology, Guangzhou Medical University, Guangzhou, China

**Keywords:** bone metabolism, immunometabolism, mitochondrial quality control, mitochondrial transfer, osteoimmunology

## Abstract

The maintenance of bone homeostasis relies on a dynamic interplay between skeletal and immune cells. Traditional paracrine and receptor-based models cannot fully explain the reciprocal communication that underpins bone remodeling. Intercellular mitochondrial transfer refers to the movement of one or more mitochondria from a donor cell to a recipient cell. In this review, this term is distinguished from the intercellular transfer of mitochondrial components, which is described separately according to the nature of the transferred material. Mitochondrial transfer occurs via three primary pathways: direct contact, extracellular vesicles (EVs), and free mitochondrial release. Emerging evidence positions intercellular mitochondrial transfer as a novel form of organelle-level communication in the regulation of bone homeostasis. Although mitochondrial transfer has been documented extensively in bone-related pathologies, its context-dependent, bidirectional regulatory roles within the osteoimmune microenvironment have yet to be systematically elucidated. To address this gap, we systematically summarize the molecular mechanisms governing mitochondrial transfer within the osteoimmune microenvironment, with a particular emphasis on the bidirectional communication network between skeletal cells and immune cells. We further discuss its dual pathophysiological roles in bone-related disorders and highlight emerging therapeutic strategies based on mitochondrial transfer. We also propose a novel framework for bone remodeling interventions that centers on precise control of both transfer directionality and mitochondrial quality. This conceptual integration provides a theoretical foundation and translational guidance for precision regenerative medicine and immunometabolic therapies in bone-related diseases.

## 1 Introduction

The homeostasis of bone metabolism relies on dynamic crosstalk between skeletal and immune cells. Bone marrow mesenchymal stem cells (BMSCs), osteoblasts, osteocytes, and chondrocytes interact closely with macrophages, osteoclast precursors, lymphocytes, and other immune populations to balance bone formation, resorption, and repair (1). This coordination is mediated by canonical osteoimmune pathways, as well as pro-inflammatory cytokines and extracellular vesicles (EVs) (2, 3). Immune cells guide matrix remodeling, while skeletal cells in turn orchestrate immune regulation (4). Collectively, these findings position osteoimmunity not as a cytokine-centric signaling cascade, but as an integrated cellular, molecular, and metabolic network that operates within the bone microenvironment.

Intercellular mitochondrial transfer adds an organelle-level dimension to this network, extending the reach of osteoimmune communication beyond the soluble factor paradigm (5, 6). Intercellular mitochondrial transfer refers to the movement of one or more mitochondria from a donor cell to a recipient cell. This definition follows the recent consensus framework and specifically distinguishes the transfer of whole mitochondria from the intercellular trafficking of mitochondrial components. The latter process may occur independently of whole-mitochondrion transfer and are therefore described separately in this review according to the nature of the transferred cargo (7). In the bone microenvironment, this process is bidirectional: skeletal cells can donate mitochondria to immune cells, while immune cells can reciprocally transfer mitochondria to skeletal cells (8). Mitochondrial transfer occurs via three distinct routes: direct contact, EVs, and free mitochondrial release. Cell-to-cell connections with direct membrane continuity enable the transport of intact mitochondria along cytoskeletal tracks (9). EVs package mitochondria, mitochondrial fragments, or mitochondrial DNA (mtDNA) for delivery to recipient cells (10, 11). Mitochondria can be released as free organelles and subsequently internalized by neighboring cells (12).

The functional outcome of mitochondrial transfer is not determined solely by its occurrence, but by a convergence of determinants: donor mitochondrial quality, transfer route, recipient cell state, and microenvironmental context. Existing reviews have primarily addressed the metabolic rescue conferred by mitochondrial transfer, whereas the pathological propagation driven by damaged mitochondria has remained underexplored. However, the delivery of functional mitochondria can restore oxidative phosphorylation (OXPHOS), adenosine triphosphate (ATP) production, and redox balance, thereby sustaining cell survival and differentiation (13). Conversely, the transfer or release of damaged mitochondria propagates oxidative stress and inflammatory signals. Therefore, mitochondrial transfer operates as either a homeostatic rescue mechanism or a conduit for pathological propagation, a bifurcation governed by cargo integrity and the prevailing state of the recipient microenvironment.

Accordingly, we define the osteoimmune mitochondrial transfer network as a closed-loop regulatory framework in which skeletal cells and immune cells act as both donors and recipients, enabling a bidirectional exchange of mitochondria. Biophysical and inflammatory cues from the bone microenvironment are sensed by these cells, encoded into mitochondria-associated signals, and transmitted to the reciprocal cell type to regulate metabolic and immune homeostasis. In turn, the effector responses elicited by these mitochondrial signals can remodel the extracellular matrix, modulate osteogenic and osteoclastic activity, and reshape the local metabolic niche, thereby feeding back to the initial microenvironmental input. Thus, this network is defined by directional mitochondrial trafficking, cargo-quality-dependent signaling, and context-dependent functional outcomes, rather than representing a simple binary switch between rescue and injury.

## 2 Major pathways of mitochondrial transfer

Intercellular mitochondrial transfer can be divided into three categories, including the formation of cellular connections that permit mitochondria to move directly from one cell to another, ejection of mitochondria within EVs that are then delivered to recipient cells, and the release of free mitochondria that are subsequently captured and internalized by recipient cells (**Figure 1**).

**Figure 1:**
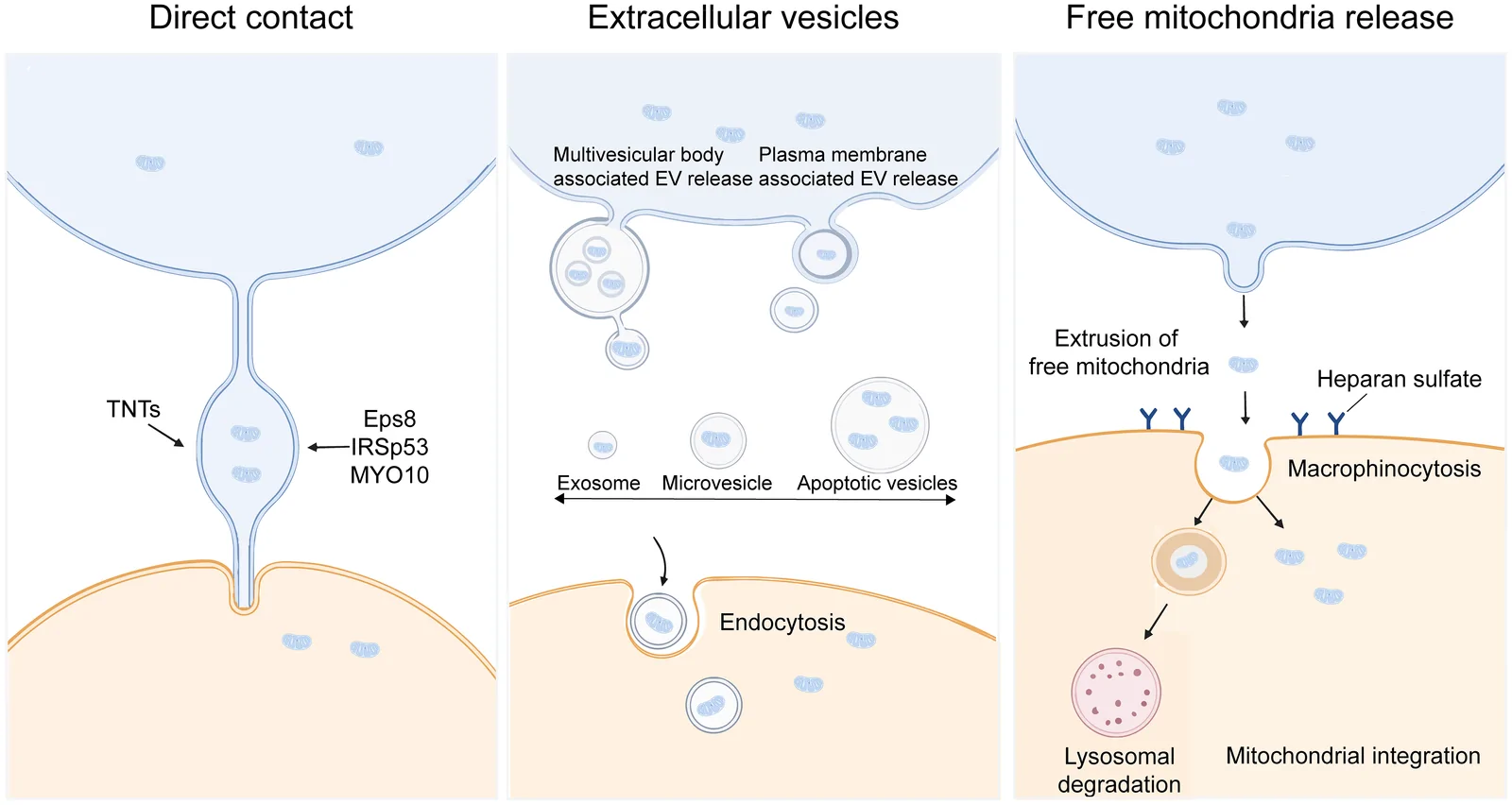
Schematic illustration of three primary modes of intercellular mitochondrial transfer and the movement of mitochondrial components. Mitochondria can be transferred via direct cell-to-cell contact mediated by tunneling nanotubes (TNTs), extracellular vesicles (EVs), or as free mitochondria released into the extracellular space. Key regulatory molecules and downstream cellular fates, including lysosomal degradation and mitochondrial integration in recipient cells, are annotated in the diagram.

### 2.1 Direct contact-mediated mitochondrial transfer

Direct contact-mediated mitochondrial transfer is achieved through three principal pathways: tunneling nanotubes (TNTs), gap junctions, and cell fusion. Among these, TNTs serve as the most prominent mechanism. They are actin-rich membrane protrusions that form transient intercellular bridges connecting donor and recipient cells (14). The biogenesis of TNTs requires the coordinated action of Eps8 and IRSp53, which regulate linear actin polymerization and membrane curvature (15). Once established, the active transport of mitochondria along TNTs is driven by the molecular motor protein MYO10, which shuttles these organelles from donor to recipient cells (16). For gap junctions, proteins such as connexin-43 (Cx43) primarily contribute to stabilizing intercellular contacts. However, whether intact mitochondria can be directly translocated through gap junction channels remains a subject of debate (17). In contrast to structured transfer, cell fusion results in the complete intermixing of cytoplasmic contents between cells, and is notably efficient for the rapid, localized delivery of functional mitochondria under stress conditions (18).

### 2.2 EV-mediated mitochondrial transfer

EVs are lipid-bilayer-enclosed extracellular particles that carry diverse biological cargo, including proteins, nucleic acids, and lipids, which can be classified as exosomes, microvesicles and apoptotic vesicles (19). According to recent consensus nomenclature, extracellular mitochondria enclosed within EVs are referred to as EV-Mito. EV-Mito are heterogeneous in both size and cargo, with reported diameters ranging from approximately 120 nm to 3-4 μm and with cargo that may include intact mitochondria or mitochondrial components (20, 21). The small GTPase Rab7 serves as a pivotal molecular switch for determining the ultimate destination of this cargo. Its GTP-bound form promotes lysosomal clearance of mitochondria, whereas its GDP-bound form diverts mitochondria into multivesicular bodies (MVBs) for subsequent EV secretion (22). When conventional mitophagy is impaired, an alternative pathway involving acid sphingomyelinase enables a switch from degradative to secretory autophagy, thereby allowing mitochondrial extrusion (23). Thus, rather than representing a single defined biogenesis pathway, EV-Mito release may involve multiple mechanisms, including MVB-associated and plasma membrane-associated vesicle formation. Following extracellular release, EV-Mito can be taken up by recipient cells, potentially facilitating local or systemic intercellular communication depending on the nature and functional integrity of the transferred mitochondrial cargo.

### 2.3 Free mitochondria release

The release and subsequent capture of free mitochondria represent another principal form of intercellular mitochondrial transfer (24). In this pathway, donor cells extrude intact mitochondria or fragments into the extracellular space, which can be internalized by recipient cells primarily via macropinocytosis or other phagocytic mechanisms. Previous studies have identified heparan sulfate, particularly its 6-O-sulfation modification, as a critical recognition motif that mediates the capture of extracellular mitochondria by recipient cells (25). Once internalized, free mitochondria are subject to two distinct intracellular fates. They may escape from the endolysosomal compartment and successfully integrate into the host mitochondrial network to restore bioenergetics. Alternatively, they are routed toward lysosomal degradation as a clearance mechanism. Notably, this uptake process is metabolically coupled to cysteine-glutathione homeostasis. Cathepsin H modulates cysteine availability and maintains glutathione levels, thereby creating a permissive redox environment that not only enhances the functionality of internalized mitochondria but also actively supports bone regeneration. Although less efficient than contact-dependent transfer, free mitochondrial release enables long-range delivery and can be harnessed for therapeutic mitochondrial transplantation in bone-related diseases (26).

## 3 Bidirectional crosstalk of mitochondrial transfer within bone

Bone is a complex tissue composed of skeletal and immune cells that integrates metabolic and immune homeostasis (27, 28). Intercellular mitochondrial transfer transcends its conventional role as unidirectional energetic rescue and operates instead as a bidirectional communication network, the direction and outcome of which are dynamically modulated by the local microenvironment (8). Importantly, the biological consequences of this bidirectional communication are not determined solely by the direction of transfer, but also by the characteristics of the transferred mitochondrial cargo and the local cellular context. Intercellular mitochondrial transfer and the movement of mitochondrial components between skeletal and immune cells serve not merely as metabolic compensation, but as a fundamental mode of cell-to-cell signaling at the osteoimmune interface (**Figure 2**).

**Figure 2:**
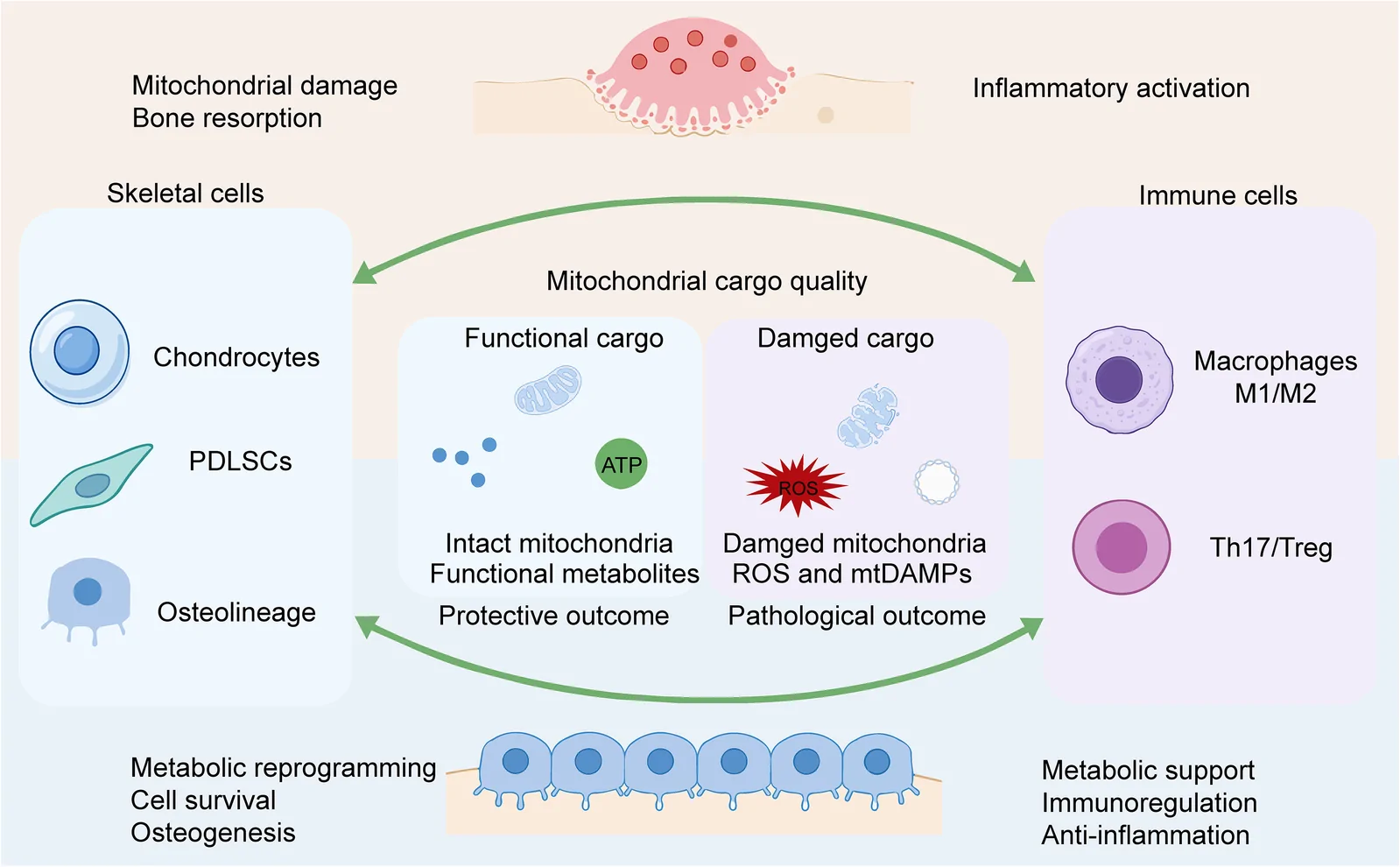
Bidirectional osteoimmunological crosstalk mediated by intercellular mitochondrial transfer and the movement of mitochondrial components in bone homeostasis. Pathological mitochondrial cargo (reactive oxygen species (ROS)-enriched damaged mitochondria) drives inflammatory immune cell activation, osteoclast-dominated bone resorption and skeletal cell dysfunction. By contrast, protective mitochondrial cargo (intact functional mitochondria and ATP) supports regulatory immune cell phenotypes and potentiate osteogenic differentiation for bone regeneration.

### 3.1 Mitochondrial transfer from skeletal cells to immune cells

Mitochondrial transfer from skeletal cells to immune cells can exert either protective or pathological effects, depending on the transferred mitochondrial cargo and the surrounding cellular and microenvironmental context.

Osteolineage cells can transfer mitochondria to myeloid osteoclast precursors, thereby improving glutathione metabolism in recipient cells, suppressing ferroptosis, and attenuating bone resorption (29). Mesenchymal stem cells (MSCs) can also eliminate dysfunctional mitochondria through extracellular routes as a quality-control mechanism. Under oxidative stress, MSCs release depolarized mitochondria via ARRDC1-mediated EV-Mito that are phagocytosed by CD86^+^ macrophages and fuse with intracellular compartments to enhance macrophage bioenergetics (10). BMSCs transfer mitochondria to T helper 17 cells (Th17 cells) to reprogram the metabolic state of Th17 cells, suppress their production of pro-inflammatory cytokine interleukin-17 (IL-17), and promote their conversion toward regulatory T cells, thus exerting an immunosuppressive effect to alleviate local inflammation (30). Collectively, these findings support the notion that MSCs in the subchondral bone marrow, given their anatomical proximity and accessibility, represent a promising donor cell source for such therapeutic approaches (31).

In addition, periodontal ligament stem cells (PDLSCs) tend to modulate local inflammation, alleviate periodontitis-induced tissue destruction, and promote tissue repair (32, 33). Aquaporin 1 (AQP1)-overexpressing mitochondria from PDLSCs can be transferred to inducible nitric oxide synthase (iNOS^+^)/CD86^+^ M1-like macrophages, promoting SigmaR1 expression and endoplasmic reticulum (ER)-mitochondria contacts. These signaling events subsequently inhibit RANKL-driven osteoclastogenesis and bone resorption while polarizing macrophages toward an arginase 1 (Arg1)^+^, CD206^+^ M2 phenotype, ultimately mitigating periodontal immunoinflammation (34).

In response to mechanical stress and local tissue injury, tissue cells transfer destructive mitochondrial components to immune cells to accelerate tissue destruction. Mechanically compressed periodontal ligament fibroblasts secrete EV-Mito, which can be taken up by macrophages and activate the NLR family pyrin domain containing 3 (NLRP3) inflammasome, leading to caspase-1-dependent pyroptosis. This pyroptotic cell death amplifies local inflammatory signals and ultimately drives alveolar bone remodeling during orthodontic tooth movement (35).

### 3.2 Mitochondrial transfer from immune cells to skeletal cells

Conversely, mitochondrial transfer from immune cells to skeletal cells can also exert context-dependent protective or pathological effects, depending on the characteristics of the transferred mitochondrial cargo and the state of recipient cells.

Inflammatory macrophages transfer EV-Mito to BMSCs, impairing osteogenic differentiation and exacerbating alveolar bone loss (36). Aberrant accumulation of succinate dehydrogenase in CD86^+^ macrophages sustains their inflammatory phenotype and aggravates mitochondrial injury, which can be propagated to osteoblasts via intercellular mitochondrial transfer, impairing their osteogenic function and exacerbating bone loss (37). M2-like macrophages can also transfer functional mitochondria to injured PDLSCs to reprogram their bioenergetic state and enhance cell survival under inflammatory conditions via activation of the p38-mitogen-activated protein kinase (MAPK) signaling pathway. Therefore, promoting beneficial mitochondrial transfer between PDLSCs and macrophages may serve as a promising therapeutic strategy for periodontal tissue regeneration (38). Mechanical force activates dynamin-related protein 1 (Drp1) in macrophages to induce mitochondrial fission, and promotes the transfer of mitochondria to BMSCs via EV-Mito through the CD200/CD200R axis, thereby enhancing the osteogenic differentiation of BMSCs and promoting bone formation (39).

### 3.3 Feedback regulation at the osteoimmune interface

Overall, the evidence summarized above indicates that bidirectional mitochondrial communication between skeletal and immune cells is highly context-dependent. Neither the direction of transfer nor the cellular source alone determines its biological outcome. Instead, the quality and functional state of the transferred mitochondrial cargo, together with the metabolic state of recipient cells and the surrounding microenvironment, collectively shape the resulting response. Functionally competent mitochondrial cargo may support metabolic homeostasis, cellular survival, and tissue repair, whereas damaged mitochondrial cargo or mitochondria-derived danger signals may promote inflammatory activation and tissue damage. Thus, bidirectional mitochondrial communication can contribute to either adaptive or maladaptive feedback within the osteoimmune microenvironment, providing a conceptual basis for understanding its diverse roles in bone homeostasis and disease.

## 4 Determinants of mitochondrial transfer and functional outcomes

The ultimate outcome of intercellular mitochondrial trafficking, whether metabolic repair or inflammatory amplification, is dictated by four integrated determinants: external environmental stress, donor cargo quality, carrier shielding condition and host cell clearance capacity (**Figure 3**).

**Figure 3:**
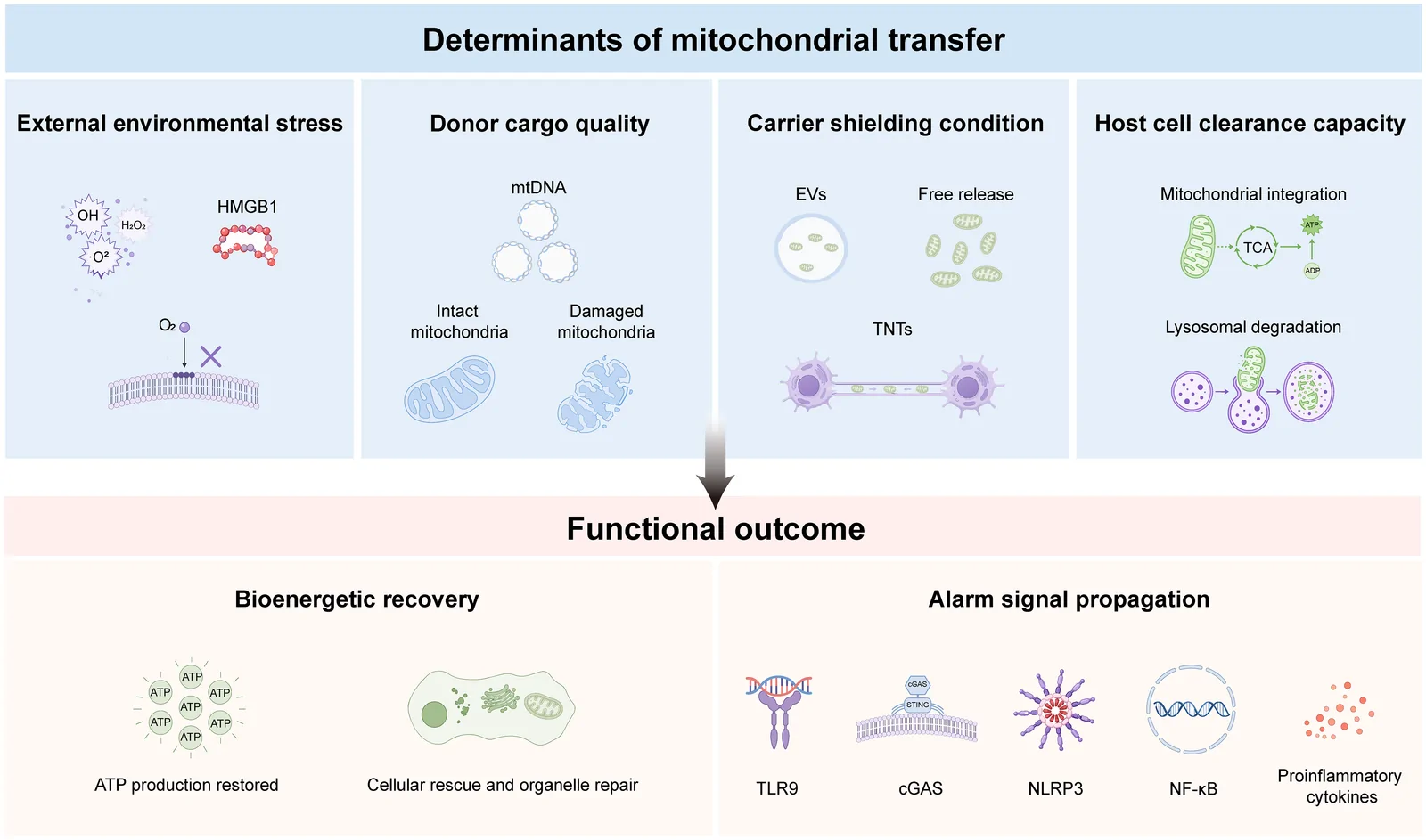
Core determinants governing the functional outcomes of intercellular mitochondrial transfer. External environmental stress, donor cargo quality, carrier shielding condition, and host cell clearance capacity collectively determine whether mitochondrial transfer leads to bioenergetic cellular rescue or alarm signal propagation and innate-immune-related inflammatory cascades.

Factors including reactive oxygen species (ROS), hypoxia, inflammatory mediators, and Ca²^+^ homeostasis disruption can modulate cytoskeletal remodeling, mitochondrial dynamics, and intercellular communication, thereby affecting the likelihood of mitochondrial transfer (40, 41). Beyond these external cues, the intrinsic properties of donor mitochondria serve as critical determinants. The membrane potential, oxidative damage burden, cristae integrity, and respiratory capacity of donor mitochondria critically determine whether the transferred organelles confer metabolic benefits or provoke inflammatory responses in recipient cells (42, 43). Functional mitochondrial transfer restores oxidative phosphorylation and energy metabolism, whereas damaged mitochondrial components preferentially activate inflammasomes and other innate immune pathways (44).

In addition to the quality of the mitochondrial cargo itself, the integrity of the transport carriers plays a decisive role in shaping the functional outcome. TNT-mediated direct transfer and EV-encapsulated transport limit the exposure of mitochondrial cargo to the extracellular environment. Conversely, oxidized mtDNA, cardiolipin, and other damaged mitochondrial components released into the extracellular space are more prone to act as mitochondrial damage-associated molecular patterns (mtDAMPs) and trigger innate immune responses (45, 46). Thus, the integrity of transport carriers and the quality of their cargo collectively determine whether mitochondria-associated communication favors metabolic repair or inflammatory amplification.

Once the mitochondrial cargo reaches the recipient cell, its ultimate fate depends on the metabolic and lysosomal processing capacity of the host cell. Following internalization of exogenous mitochondria or mitochondrial cargo, recipient cells may display diverse responses, including integration into oxidative phosphorylation, metabolic reprogramming, lysosomal degradation and recycling, or activation of inflammatory signaling. The intrinsic metabolic state, mitochondrial quality control machinery, and lysosomal processing capacity of recipient cells are key determinants of how incoming mitochondrial cargo is handled (47–49).

## 5 Pathological roles of mitochondrial transfer in bone-related diseases

As a novel organelle-level mode of intercellular communication, mitochondrial transfer plays a pivotal role in maintaining metabolic homeostasis at the osteoimmune interface (50, 51). However, under conditions of chronic inflammation, oxidative stress, mechanical overload, or aging-related insults, defects in donor cell mitochondrial quality control, impairment of intercellular transport routes, or aberrant processing of transferred mitochondria by recipient cells can collectively drive pathological remodeling of the bidirectional mitochondrial communication between skeletal and immune cells (52, 53). These alterations provide a common mechanistic basis through which dysregulated mitochondrial communication may contribute to the onset and progression of multiple bone-related diseases (**Figure 4**).

**Figure 4:**
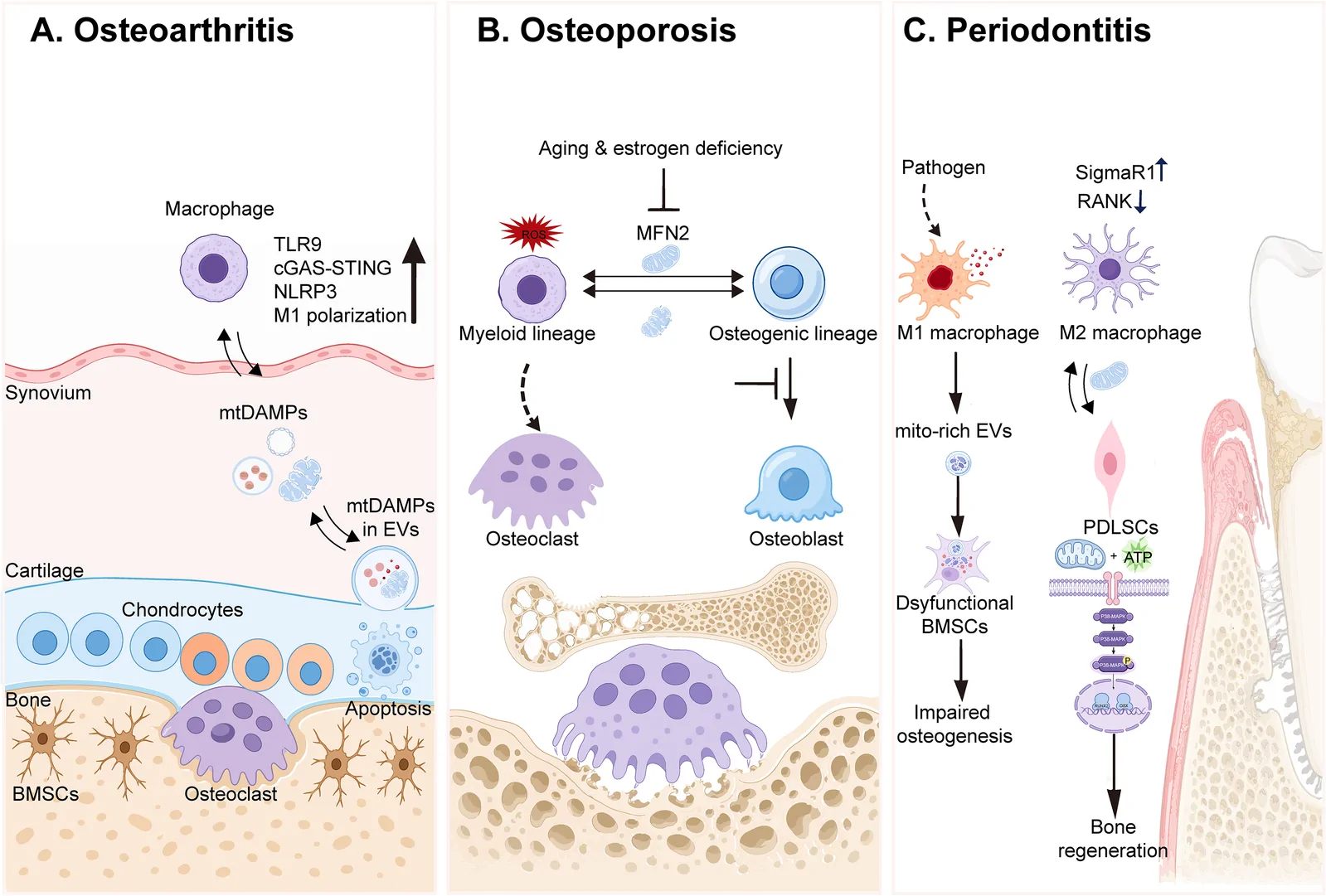
Mitochondrial transfer-related pathological events in representative bone-related disorders. **(A)** Osteoarthritis: mitochondrial damage-associated molecular patterns (mtDAMPs) derived from apoptotic chondrocytes trigger macrophage-mediated inflammatory responses within synovium and cartilage. **(B)** Osteoporosis: aging- or estrogen-deficiency-driven mitochondrial dysfunction disturbs the crosstalk between myeloid and osteogenic lineage cells. **(C)** Periodontitis: mitochondria-rich extracellular vesicles shift macrophage polarization and modify the osteogenic capacity of periodontal ligament stem cells (PDLSCs), ultimately altering alveolar bone remodeling.

Among these, osteoarthritis, osteoporosis, and periodontitis share a common pathological feature: chronic inflammation drives bone remodeling imbalance, underpinned by collapsed mitochondrial quality control between immune and mesenchymal cells, ultimately leading to oxidative stress, premature senescence, and exhausted regeneration (54–58). Mechanistically, pathological mitochondrial cargo skew recipient immune cells toward a glycolytic, pro-inflammatory phenotype, which converges on impaired osteogenesis, enhanced osteoclastogenesis, and cartilage degeneration (59–61). The dysregulation of mitochondrial transfer follows conserved principles across these diseases, while each condition simultaneously manifests features uniquely shaped by its specific cellular and inflammatory milieu.

### 5.1 Osteoarthritis

Osteoarthritis is a whole-joint disorder characterized by cartilage degeneration, synovial inflammation, and aberrant subchondral bone remodeling (62, 63). Within the osteoarthritis pathological microenvironment, mitochondrial transfer and metabolic communication are substantially dysregulated within the joint, which is primarily composed of chondrocytes and immune cells (52, 60).

Chondrocytes reside in a physiologically hypoxic environment and thus rely heavily on efficient mitochondrial quality control mechanisms (64). Inflammatory stimulation, oxidative stress, and abnormal mechanical loading disrupt chondrocyte metabolic homeostasis, leading to redox imbalance, insufficient energy production, and cellular dysfunction, which collectively promote cartilage degeneration (65). Damaged mitochondria and mitochondrial fragments from chondrocytes accumulate in the synovial compartment and drive inflammation (66). After uptake by synovial resident macrophages, the pathological mitochondrial cargo may activate Toll-like receptor 9 (TLR9), cyclic GMP-AMP synthase–stimulator of interferon genes (cGAS-STING), and NLRP3 inflammasome pathways, promoting M1-like polarization and the release of tumor necrosis factor alpha (TNF-α), IL-1β, and matrix metalloproteinases (MMPs), thereby accelerating cartilage degradation (67, 68). In turn, M1-like macrophages release EV-Mito, which amplify oxidative stress and drive mitochondrial dysfunction and apoptosis in chondrocytes, further exacerbating osteoarthritis progression (69, 70).

### 5.2 Osteoporosis

Osteoporosis is characterized by an imbalance between bone formation and bone resorption (71–73). Under physiological conditions, osteogenic lineage cells can transfer mitochondria to myeloid precursor cells, which in turn inhibit osteoclast activity (29). Receptor-interacting serine/threonine-protein kinase 4 (RIPK4), a protein kinase involved in mitochondrial dynamics, regulates osteogenesis through the mitofusin 2 (MFN2)-mediated control of mitochondrial dynamics. Interfering with RIPK4 in osteolineage cells disrupts mitochondrial transfer to myeloid cells, thereby contributing to the development of osteoporosis (74). In the aged bone marrow microenvironment, CD206^+^ M2-like macrophages exhibit age-related dysfunction with impaired mitochondrial integrity. The capacity of CD206^+^ M2-like macrophages to act as mitochondrial donors for neighboring cells is constrained by their own bioenergetic failure, thereby limiting their potential to support stem cell function (75). Dysfunctional CD80^+^ F4/80^+^ M1-like macrophages transfer damaged mitochondria to osteoblasts and BMSCs, impairing their function and exacerbating bone resorption (76).

Together, these events establish a self-reinforcing vicious cycle, characterized by diminished bone formation and excessive osteoclast activity, which ultimately exacerbates osteoporotic progression, accompanied by progressive skeletal fragility and microarchitectural deterioration (77).

### 5.3 Periodontitis

Periodontitis is a chronic inflammatory disease driven by periodontal pathogens, ultimately leading to alveolar bone resorption and tooth loss (78, 79). Dysregulated mitochondrial communication between PDLSCs and locally infiltrating immune cells constitutes a major barrier to effective *in situ* periodontal tissue regeneration (80–82). Following pathogen infiltration, PDLSCs and local macrophages undergo severe mitochondrial oxidative stress and dynamic disruption, including excessive fission and depolarization, which substantially compromise intracellular mitochondrial quality (83).

In periodontitis, dysfunctional macrophages release EV-Mito to BMSCs, disrupting their mitochondrial dynamics and suppressing osteogenic differentiation via the lipocalin 2 (LCN2)/metalloendopeptidase OMA1 (OMA1)/optic atrophy 1 (OPA1) pathway, thereby aggravating alveolar bone loss (36). Whether dysfunctional mitochondria from periodontal cells can be directly transmitted to adjacent immune cells within the periodontal niche remains to be elucidated. However, evidence from other bone-related diseases supports the pathogenic relevance of such intercellular mitochondrial communication. In periodontal bone remodeling, mechanical compression can induce periodontal fibroblasts to secrete EV-Mito, which can activate the NLRP3 inflammasome in macrophages and trigger pyroptosis, thereby amplifying local inflammation and promoting alveolar bone remodeling (35).

## 6 Therapeutic strategies targeting mitochondrial transfer

The pathological shift of mitochondrial transfer from protective metabolic rescue to inflammatory amplification underpins the progression of multiple bone-related diseases. Targeting this organelle-level communication axis, rather than conventional cytokine or receptor interventions, offers a novel paradigm for restoring osteoimmune homeostasis. The controlled modulation of mitochondrial transfer, particularly in terms of directionality and cargo quality, has emerged as a promising therapeutic frontier. Based on current mechanistic insights, we propose that therapeutic intervention can be categorized into four principal dimensions: enhancement of endogenous protective transfer, blockade of pathological mitochondrial propagation, exogenous mitochondrial delivery, and engineered mitochondrial transfer (**Figure 5**).

**Figure 5:**
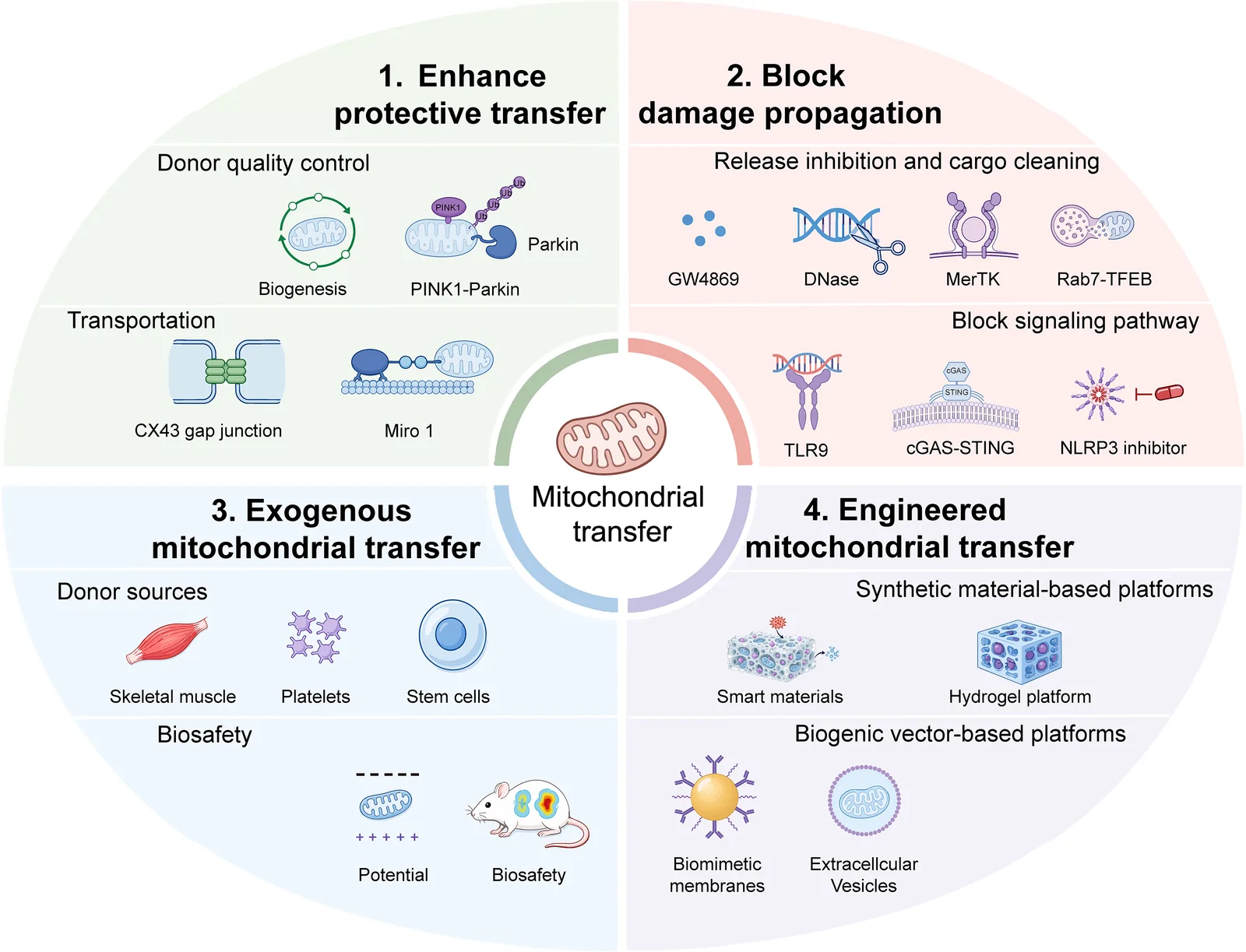
Four major therapeutic strategies targeting intercellular mitochondrial transfer for bone-related diseases (1). Enhancement of endogenous protective mitochondrial transfer via donor quality control and optimized transportation machinery (2); Blockade of pathological damage propagation through cargo clearance, inhibition of release and suppression of inflammatory signaling (3); Exogenous mitochondrial transfer covering diverse donor sources together with biosafety considerations (4); Engineered mitochondrial transfer based on synthetic material platforms and biogenic vector systems. Key small-molecule agents, protein regulators and biomaterial carriers are summarized in each module.

### 6.1 Enhancement of endogenous protective mitochondrial transfer

Strategies aimed at potentiating endogenous mitochondrial transfer ultimately converge on two interrelated goals: expanding the mitochondrial reservoir within donor cells and facilitating the efficiency of intercellular transportation. The therapeutic net gain hinges on how well these two dimensions are concurrently optimized. With this foundation, measures to upgrade mitochondrial quality can then take effect.

A prerequisite for any such intervention is that the donor mitochondria themselves must be functionally competent. Hence, donor cells are preferably maintained at low passage and in a non-senescent state, preserving adequate membrane potential, oxygen consumption, ATP yield, and controlled ROS levels (84, 85). For instance, in osteoporotic settings, konjac glucomannan -polyethylene glycol-functionalized superparamagnetic iron oxide nanoparticles have been shown to rejuvenate aged macrophages by activating autophagy and promoting Fe-S cluster biogenesis, thereby improving mitochondrial integrity. Meanwhile, these nanoparticles steer macrophages toward an M2 phenotype, rendering them more compatible with the oxidative and inflammatory milieu characteristic of senescent bone marrow stromal cells. The resulting high-grade mitochondria are subsequently transferred through Cx43-dependent gap junctions to recipient cells, restoring their bioenergetic fitness and osteogenic differentiation, and ultimately fostering bone regeneration in aged osteoporotic models (86).

Parallel efforts have been directed at boosting the transfer machinery itself. Genetic engineering of mesenchymal stem cells to overexpress CD38, for example, triggers the CD38/inositol 1,4,5-trisphosphate receptor (IP3R)/Ca²^+^ signaling cascade. The ensuing rise in mitochondrial calcium promotes fusion of mitochondria-enriched vesicles with the plasma membrane, leading to a threefold increase in the release of functional mitochondrial extracellular vesicles. These vesicles are capable of delivering intact mitochondria to recipient cells, correcting mtDNA aberrations and ameliorating disease progression, as demonstrated in a murine model of Leber hereditary optic neuropathy (87).

Despite these encouraging observations, robust direct evidence that physicochemical preconditioning or pharmacological modulation consistently amplifies therapeutic efficacy remains scarce. Protocol refinement and rigorous safety validation are still needed. Moreover, the success of these approaches is inherently contingent on the quality of the donor mitochondrial pool, as noted earlier. Looking ahead, a balanced modulation of mitochondrial turnover, enhancing PTEN-induced kinase 1 (PINK1)-Parkin-mediated mitophagy to clear depolarized organelles, while simultaneously activating the AMP-activated protein kinase (AMPK)–sirtuin 1 (SIRT1)–peroxisome proliferator-activated receptor gamma coactivator 1-alpha (PGC-1α) axis to sustain biogenesis may offer a complementary strategy to augment the available mitochondrial reserve and potentiate the benefits of transfer-oriented therapies (88–90).

### 6.2 Blockade of pathological mitochondrial propagation

Pathological mitochondrial propagation intensifies local inflammation and drives tissue destruction through the release of mtDAMPs. Intervening in this process can be framed around two interconnected priorities: limiting the generation and persistence of noxious mitochondrial constituents, and interrupting the inflammatory cascades they set in motion.

At the level of damage control, efforts are directed toward reducing the release of mtDAMP-bearing vesicles and facilitating their clearance once liberated. Inhibition of exosome biogenesis or release, for instance, with GW4869, has been shown to curtail the spread of mtDNA-laden vesicles, as evidenced in orthodontic tooth movement, where mechanically strained periodontal ligament fibroblasts secrete exosomal mtDNA that triggers macrophage NLRP3-dependent pyroptosis (35). Complementary to this, extracellular mtDNA can be degraded by deoxyribonuclease I (DNase I), while phagocytic uptake via Mer receptor tyrosine kinase (MerTK) and lysosomal degradation orchestrated by Ras-related protein Rab-7 (Rab7) and transcription factor EB (TFEB) represent endogenous clearance routes for mitochondrial fragments and debris (91–93). Antioxidant intervention, such as N-acetylcysteine supplementation, further reduces oxidative stress and thereby limits the production of oxidized mtDNA, a potent inflammasome agonist. In addition, directly hindering the intercellular transfer of mitochondria to recipient cells has proven equally effective: dihydrobaicalein disrupts the transmission of functional mitochondria from BMSCs to osteoclast precursors, concurrently suppressing receptor activator of nuclear factor κB (RANK)-receptor activator of nuclear factor κB ligand (RANKL) signaling, which prevents osteoclast overactivation in postmenopausal osteoporosis (94). Similarly, pharmacological blockade of sphingosine kinase 1 (SphK1) inhibits cementocyte-to-osteoclast mitochondrial transfer and alleviates orthodontic root resorption (95).

When upstream clearance is insufficient to contain mtDAMP burden, downstream signaling pathways become the primary therapeutic target. Incomplete mtDNA degradation activates TLR9 in endosomal compartments and engages the cGAS-STING-interferon regulatory factor 3 (IRF3) axis to elicit type I interferon responses, whereas oxidized mtDNA promotes NLRP3 inflammasome assembly and IL-1β/IL-18 maturation. Pharmacological antagonists targeting TLR9, cGAS/STING, or NLRP3 (e.g., MCC950) have shown promise in attenuating mtDAMP-driven inflammation (96–99). For example, in osteoarthritis, dysfunctional nudix hydrolase 8 (Nudt8) in chondrocytes leads to cytosolic mtDNA accumulation and subsequent cGAS-STING activation, and either targeting Nudt8 or supplementing pantethine can interrupt this deleterious cascade (100). In the orthodontic model, genetic ablation of Nlrp3 or Caspase1/11 abrogates pyroptosis and moderates alveolar bone remodeling, providing a rationale for precise modulation of tooth-movement rate (35).

Taken together, a rational intervention strategy should follow a hierarchical principle, prioritizing the containment and elimination of damage signals before resorting to signal blockade, while recognizing that the optimal sequence and combination of these approaches await further validation in specific pathological contexts (101, 102).

### 6.3 Exogenous mitochondrial transfer

In contrast to strategies that rely on endogenous modulation of donor cells, exogenous mitochondrial transfer adopts a more direct approach, which supplies isolated, functionally competent mitochondria directly to diseased tissues, can be seen as mitochondrial transplantation. Specifically, mitochondrial transplantation refers to a procedure in which isolated mitochondria are administered to a recipient with the intent of eliciting a therapeutic benefit. This off-the-shelf rescue strategy offers the advantage of immediate bioenergetic restoration, making it particularly attractive for acute or rapidly progressive conditions where time is of the essence.

Skeletal muscle serves as the preferred source of exogenous mitochondria, attributable to its favorable immunogenicity profile and the absence of tumorigenic risk otherwise associated with whole-cell transplantation (103, 104). Alternative sources, including platelets and mesenchymal stem cells, are under active investigation but require further validation (105, 106). Regardless of origin, the therapeutic rationale hinges on the ability of exogenous mitochondria to be internalized by recipient cells and restore oxidative phosphorylation, ATP production, and redox balance, while concurrently suppressing apoptotic and inflammatory cascades.

Several disease models have demonstrated the translational potential of exogenous mitochondrial transfer. In osteoarthritis, mitochondria derived from bone marrow or umbilical cord MSCs, when transferred to osteoarthritic chondrocytes, have been shown to inhibit apoptosis, restore ATP levels, alleviate inflammation and oxidative stress, and upregulate type II collagen expression, thereby preserving chondrocyte function and retarding cartilage degeneration (44, 107). Further refinements in culture conditions have enabled the generation of mitochondria with enhanced bioenergetic profiles. For instance, mitochondria from human BMSCs cultured in optimized medium exhibit higher yield and ATP output, and activate AMPK/mitochondrial transcription factor A (TFAM)-driven biogenesis in recipient chondrocytes while curbing energetically costly processes; intra-articular delivery of these mitochondria effectively promotes cartilage repair (108). In the periodontal setting, mitochondria isolated from human embryonic stem cells have been transferred to gingival fibroblasts, resulting in elevated ATP levels, reduced oxidative stress, and suppression of IL-6 and TNF-α expression, thus mitigating local inflammatory damage (109).

However, the direct injection nature of this strategy places exceptionally high demands on mitochondrial quality. Isolation procedures, typically differential or density gradient centrifugation, must be coupled with rigorous quality control encompassing structural integrity (electron microscopy), functional competence (membrane potential, oxygen consumption rate, ATP production), and biosafety (verification of mitochondrial markers, exclusion of non-mitochondrial contaminants, sterility, and endotoxin testing) (21, 110, 111). Without such safeguards, the introduction of dysfunctional or damaged mitochondria could paradoxically exacerbate inflammatory signaling. Therefore, while exogenous mitochondrial transfer holds considerable therapeutic promise, its successful clinical translation will depend as much on stringent quality assurance as on the biological efficacy of the mitochondria themselves.

### 6.4 Engineered mitochondrial transfer

Once isolated from their native intracellular environment, exogenous mitochondria become highly susceptible to temperature fluctuations, osmolarity shifts, calcium overload, ROS, and mechanical shear stress. Direct injection, though procedurally straightforward, suffers from rapid diffusion, swift clearance, and precipitous functional decay, with only a small fraction ultimately internalized by target cells. Engineered mitochondrial delivery has shown promising therapeutic potential in inflammatory and degenerative disorders, although its application to bone-related diseases remains at an early stage (**Table 1**).

**Table 1:** Representative studies of engineered mitochondrial transfer in bone-related diseases.

Disease model	Donor cells	Engineered method	Recipient cells	Functional outcome	Delivery dose	Administration route	Evidence of intact mitochondrial function	Safety assessment	Reference
Bone defect (mouse)	Macrophages	GAD/Ag-pIO 3D hydrogel scaffold	BMSCs	Accelerates bone regeneration	N/A	Local application	Reduced ROS and elevated ATP level	Excellent biocompatibility	(151)
Bone defect (mouse)	Macrophages	Gel@MDI hierarchical hydrogel	BMSCs	Enhances osteogenic differentiation	N/A	Local application	Reduced ROS and elevated ATP level	Excellent biocompatibility	(114)
Bone fracture (rat)	BMSCs	Mito@ACs artificial cell microspheres	BMSCs	Ameliorates senescent phenotype of aged BMSCs	15mg *in vivo*	Local application	Elevated ATP level and membrane potential	Excellent biocompatibility	(152)
Osteoporosis (mouse)	Macrophages	CXCR4-engineered	BMSCs	Enhances osteogenic differentiation	N/A	Intravenous injection	Reduced ROS and elevated ATP level	Excellent biocompatibility	(130)
Osteoporosis (rat)	Macrophages	Cerium-based nanosystem	BMSCs	Enhances bone formation	10ug/ml *in vitro*	Local application	Restored membrane potential and reduced ROS	Excellent biocompatibility	(75)
Osteoporosis (rat)	Macrophages	Functionalized Fe_3_O_4_ nanoparticles	BMSCs	Enhances osteogenic differentiation	N/A	Local application	Restored membrane potential and calcium homeostasis	Excellent biocompatibility	(86)
Rheumatoid arthritis (mouse)	BMSCs	Folate-modified macrophage membrane encapsulating mitochondria and AUTAC4	Macrophages	Alleviates synovial inflammation and bone destruction	40 μM *in vitro*	Targeted delivery	Restored mitophagy	Excellent biocompatibility	(153)
Periodontitis (mouse)	PDLSCs	AQP1-overexpressing mitochondria	Macrophages	Attenuates bone loss by regulating mitochondria-ER contacts	25 μg *in vivo*	Local application	Alleviated oxidative stress	Excellent biocompatibility	(34)

Synthetic material-based platforms encompass hydrogel-based systems and smart responsive materials, both of which rely on artificially fabricated abiotic materials to preserve mitochondrial integrity and enable spatiotemporally controlled delivery (112). Hydrogel platforms represent a major avenue for achieving sustained local delivery. Mitochondria encapsulated in gelatin-based hydrogels retain respiratory and ATP-generating activity, providing a continuous energy supply for energy-demanding regenerative processes (113). A more sophisticated design is Gel@MDI, an engineered hierarchical hydrogel that responds to the acidic bone injury microenvironment. This system incorporates macrophage-targeted nanogels that upregulate mitochondrial Rho GTPase 1 (Miro1) expression, thereby promoting efficient mitochondrial transfer from macrophages to recipient BMSCs. The transferred mitochondria restore ATP production and oxidative phosphorylation in BMSCs, enhance their osteogenic differentiation capacity, and ultimately achieve bone regeneration through coordinated immunomodulation and metabolic reprogramming (114). Smart materials are designed to perceive and respond to specific pathophysiological signals, enabling targeted accumulation and on-demand cargo release. Surface modification with alendronate or acidic-amino-acid-rich peptides such as aspartate octapeptide (Asp8) promotes binding to bone hydroxyapatite, thereby augmenting mitochondrial localization at sites of active remodeling. Concurrently, the introduction of ROS-sensitive linkers enables preferential cargo release in highly inflamed pathological niches, improving spatiotemporal delivery specificity (115–117). A recent paradigm is the pH-responsive pH-low insertion peptides (pHLIPs)–PEG–triphenylphosphonium (TPP)–mitochondria complex, which exploits acidic inflammation via a pH-triggered decoupling strategy, enabling self-stripping and efficient mitochondrial delivery to CD86^+^ M1-like macrophages. The transferred mitochondria restore cellular bioenergetics and drive M2 phenotypic reprogramming, effectively alleviating periodontal inflammation (118).

Biogenic vector-based platforms encompass biomimetic membrane encapsulation, EV-based carriers, and genetically engineered cell vehicles. Unlike their synthetic counterparts, these biogenic vectors leverage the intrinsic biointerfacial properties derived from their cellular origin, including homing receptors, adhesion molecules, and immunomodulatory ligands, to achieve active targeting, immune evasion, and efficient cellular internalization. Biomimetic membranes and EV carriers offer physical protection for mitochondrial cargo during delivery. Encapsulating isolated mitochondria within natural cell membranes, or generating microvesicles enriched in functionally competent mitochondria, represents a promising strategy to shield mitochondria from extracellular stressors while preserving their bioactivity (119–121). Erythrocyte-based mitochondrial carriers, for instance, co-deliver functional mitochondria and oxygen to macrophages in inflammatory microenvironments. This delivery reprograms macrophage metabolism, suppresses osteoclast differentiation, and promotes M2 phenotypic switching to alleviate inflammation-associated bone destruction (122). Adipose-derived stem cells can transfer EV-Mito, activating the MAPK/Extracellular signal-regulated kinase 1/2 (ERK1/2)/Forkhead box O1 (FoxO1)/autophagy pathway and inducing metabolic reprogramming in dendritic cells, thereby offering a novel therapeutic strategy for temporomandibular joint osteoarthritis (123). These EV-based approaches can also be integrated with hydrogel platforms to achieve sustained local delivery. For example, adipose-derived stem cell derived EV-Mito, when incorporated into hydrogels, have been shown to promote M2-like macrophage polarization and accelerate wound healing (124). Genetic modification further refines targeting precision and facilitates internalization. Exogenous mitochondria may enter recipient cells via macropinocytosis. Incorporation of cell-targeting ligands or cell-penetrating peptides onto carrier surfaces, coupled with physical adjuncts such as centrifugation or magnetic attraction, can increase mitochondria-to-cell contact and promote uptake (125–129). A representative example is a live mitochondrial delivery system composed of C-X-C chemokine receptor type 4 (CXCR4)-engineered macrophages loaded with nanozyme-functionalized mitochondria, which delivers functional mitochondria to metabolically compromised BMSCs, restores mitochondrial respiration, enhances osteogenic differentiation, and alleviates oxidative apoptosis, thereby promoting metabolic recovery and bone formation in osteoporotic mice (130).

Although these engineering approaches have been extensively explored for drug and EV delivery, their application to intact, functionally competent mitochondria remains nascent (131, 132). Crucially, protective mitochondrial transfer should be preserved or enhanced through donor cell quality screening, EV-Mito purification, and modulation of trafficking pathways, while pathological propagation should be inhibited (133–135). Collectively, these strategies advocate a selective therapeutic philosophy: low-quality mitochondrial cargo should be removed or rendered immunologically inert, whereas structurally intact, functional mitochondria deserve preservation and protection.

## 7 Translational challenges and future research directions

Mitochondrial transfer provides a new perspective for understanding the pathogenesis of osteoarthritis, osteoporosis, periodontitis, and other bone-related diseases, and has shown considerable therapeutic potential (101). However, the current evidence remains largely derived from *in vitro* studies and animal models. Standardized protocols have yet to be established for mitochondrial sourcing, isolation and preparation, functional characterization, dose determination, and safety evaluation (136, 137). Successful clinical translation will require not only improvements in mitochondrial delivery efficiency but also the establishment of comprehensive evaluation systems to verify the authenticity of mitochondrial transfer, its functional efficacy, *in vivo* biodistribution, and long-term safety (138–140).

### 7.1 Evidence standards for mitochondrial transfer

The detection of mitochondrial fluorescence in recipient cells is insufficient to demonstrate the intercellular transfer of intact and functional mitochondria (41, 101). To improve comparability among studies, evidence for mitochondrial transfer may be categorized into four levels according to its evidentiary strength. Level I evidence comprises direct *in vivo* evidence. This level requires donor-specific genetic or mitochondrial labeling, together with intravital imaging, three-dimensional tissue imaging, or cell-type-specific isolation of recipient cells, to establish the directionality of mitochondrial transfer and identify the recipient-cell population (5). Level II evidence comprises direct *in vitro* evidence. This requires live cell imaging to capture the dynamic transfer of mitochondria from donor to recipient cells, combined with functional assays in recipient cells to determine whether the transfer is associated with functional recovery (42). Level III evidence comprises component-based evidence, including the detection of donor-specific mtDNA haplotypes, genetic labels, or mitochondrial proteins in purified recipient cells (141). Level IV evidence comprises indirect or inferential evidence, such as static colocalization, changes in mitochondrial fluorescence signals, or the inhibition of putative transport pathways. Although these findings may suggest the occurrence of mitochondrial transfer, they cannot, when considered in isolation, establish the intercellular transfer of intact mitochondria (101).

Experimental designs should also incorporate appropriate controls to distinguish genuine mitochondrial transfer from potential confounding processes. Such controls are important for excluding artifacts arising from the leakage or transfer of lipophilic dyes, such as MitoTracker, donor-recipient cell fusion, and the phagocytic uptake of apoptotic bodies or cellular debris (17). More importantly, the detection of donor-derived mtDNA or mitochondrial proteins should not be interpreted as direct evidence of intact mitochondrial transfer, because these components may be released in a cell-free form or disseminated through EVs (142). Accordingly, rigorous identification of mitochondrial transfer should integrate donor-specific tracing, dynamic imaging, structural assessment, and functional validation in recipient cells.

### 7.2 Quality and safety of mitochondrial transfer

The development of mitochondria as organelle therapeutics necessitates a comprehensive quality and safety framework that spans the entire product lifecycle, from mitochondrial acquisition to clinical administration (140). This framework should address four sequential stages: donor sourcing and isolation, quality control of isolated mitochondria, storage and transportation, and *in vivo* biological assessment.

At the sourcing stage, donor screening should exclude pathogenic mtDNA variants and assess mtDNA heteroplasmy. When cultured cells are used as the mitochondrial source, heteroplasmy drift and clonal selection during prolonged culture or expansion should be carefully monitored (143). The choice of donor cell type, passage number, and culture conditions directly influences the functional competence of isolated mitochondria and therefore represents a critical determinant of downstream therapeutic efficacy.

Following isolation, rigorous quality control must be established to ensure batch-to-batch consistency and product safety. Universally accepted criteria for assessing therapeutic mitochondria have yet to be established, but potential critical quality attributes include mitochondrial purity, membrane and cristae integrity, membrane potential, oxygen consumption rate, ATP-generating capacity, endotoxin levels, and residual nuclear DNA, cellular debris, or other organelles (144). Structural integrity can be assessed by examining the mitochondrial double membrane and cristae, whereas membrane potential, respiratory activity, and ATP production serve as indicators of functional potency (145, 146). Dosage should not be determined solely by total mitochondrial protein content; rather, it should also account for the number of intact mitochondria, donor cell origin, and mitochondrial respiratory activity to improve comparability across batches and studies (146, 147).

Storage and transportation represent another critical juncture at which mitochondrial quality can be compromised. Isolated mitochondria are vulnerable to temperature fluctuations, oxidative stress, mechanical damage, and prolonged ex vivo exposure, all of which may impair their membrane potential and respiratory activity (148). Therefore, storage conditions and transport protocols must be rigorously validated for mitochondrial stability and functional potency, with particular attention to temperature control, buffer composition, and the time interval from isolation to administration.

Once administered, the *in vivo* fate of exogenous mitochondria should be systematically evaluated. This includes their biodistribution, accumulation in untargeted tissues, uptake by non-target cells, and long-term persistence or clearance (136). Cell-free mtDNA released from damaged mitochondria may activate innate immunity; therefore, safety assessments should include local inflammation, systemic cytokine responses, and risks associated with repeated administration (43). Previous studies have shown that tumor cells can acquire mitochondria from neighboring cells and thereby enhance their tumorigenic properties. Accordingly, preclinical studies should assess the unintended uptake of exogenous mitochondria and their potential tumor-promoting effects (149, 150).

Future evaluation frameworks should integrate all these dimensions, including mitochondrial source, purity, structural integrity, potency, batch consistency, sterility, endotoxin burden, stability, biodistribution, immunogenicity, and long-term toxicity. Establishing the relationships between these critical quality attributes and *in vivo* efficacy and safety will be essential for defining scientifically sound release criteria and advancing clinical translation.

## 8 Discussion

The understanding of bone remodeling has evolved from a focus on isolated cellular and molecular events to the recognition of a dynamic, multi-directional regulatory network. In this review, we distinguish our framework from previous models by shifting the focus from unidirectional metabolic rescue to a bidirectional, cargo-quality-dependent communication axis at the osteoimmune interface. Specifically, rather than viewing mitochondrial transfer as a simple energetic support, we emphasize that its biological consequences are shaped by the quality and functional state of the transferred mitochondrial cargo, as well as the cellular and microenvironmental context. Moreover, by linking these context-dependent effects to the shared and disease-specific features of osteoarthritis, osteoporosis, and periodontitis, our framework provides a unified perspective on these otherwise distinct bone disorders.

Several important questions remain unresolved. Firstly, the approaches used to identify mitochondrial transfer vary substantially across studies. Detection of mitochondrial fluorescence, donor-derived proteins, or mtDNA alone does not necessarily demonstrate the transfer of structurally intact and functionally competent mitochondria. More rigorous approaches integrating donor-specific tracing, dynamic imaging, structural assessment, and functional validation are therefore needed to establish bona fide mitochondrial transfer. Secondly, the determinants of mitochondrial transfer outcomes remain incompletely understood. In particular, the relative contributions of mitochondrial quality, transfer route, recipient-cell state, and the surrounding microenvironment require further clarification. And finally, how these factors interact to determine the fate and biological consequences of transferred mitochondrial cargo remains largely unknown. Future studies should therefore move beyond confirming the occurrence of mitochondrial transfer and systematically investigate how mitochondrial quality, transfer mechanisms, recipient-cell characteristics, and microenvironmental context jointly shape the fate and functional consequences of transferred mitochondrial cargo.

This framework also has implications for therapeutic development. Rather than broadly increasing or suppressing mitochondrial transfer, future strategies may need to selectively modulate the relevant transfer process according to the disease context, mitochondrial cargo quality, and recipient-cell state. Potential approaches include enhancing beneficial mitochondrial delivery, limiting the propagation of dysfunctional mitochondrial signals, and developing controlled exogenous or engineered mitochondrial delivery systems. Translation will require standardized criteria for demonstrating genuine mitochondrial transfer, quantitative assessment of mitochondrial quality and function, and systematic evaluation of dose, biodistribution, and long-term safety. Collectively, these considerations support the development of mitochondrial transfer as a potential platform for organelle-level regulation of osteoimmune homeostasis.
